# Accurate Modeling of Working Normal Rake Angles and Working Inclination Angles of Active Cutting Edges and Application in Cutting Force Prediction

**DOI:** 10.3390/mi12101207

**Published:** 2021-10-01

**Authors:** Peng Li, Zhiyong Chang

**Affiliations:** 1 Department of Mechanical Engineering, Northwestern Polytechnical University, Xi’an 710072, China; lip973@mail.nwpu.edu.cn; 2 Institute for Aero-Engine Smart Assembly of Shaanxi Province, Xi’an 710072, China

**Keywords:** turning geometry, working normal rake angle, active cutting edges, cutting force prediction, turning, machining

## Abstract

The normal rake angle is an important geometric parameter of a turning tool, and it directly affects the accuracy of the cutting force prediction. In this study, an accurate model of the working normal rake angle (WNRA) and working inclination angle (WIA) is presented, including variation in the cutting velocity direction. The active cutting edge of the turning tool is discretized into differential elements. Based on the geometric size of the workpiece and the position of the differential elements, the cutting velocity direction of each differential element is calculated, and analytical expressions for the WNRA, WIA, and working side cutting edge angle are obtained for each differential element. The size of the workpiece is found to exert an effect on the WNRA and WIA of the turning tool. The WNRA and WIA are used to predict the cutting force. A good agreement between the predicted and experimental results from a series of turning experiments on GH4169 with different cutting parameters (cutting depth and feed rate) demonstrates that the proposed model is accurate and effective. This research provides theoretical guidelines for high-performance machining.

## 1. Introduction

Turning is a basic metal cutting technique that is widely used in the aviation, aerospace, and automobile industries. Predicting the cutting force plays an important role in reducing workpiece deformation during the turning process, improving the quality of machined parts [1,2], extending tool life [3] and reducing production cost [4]. Accurate calculation of the turning tool rake angle is required for high-precision prediction of the cutting force. The rake angle of the turning tool provides an important basis for optimizing the cutting parameters, improving the tool geometry design, and analyzing the machining stability. Many researchers have studied the rake angle in turning tools [5,6,7,8,9,10,11,12,13,14].

Merchant [5] provided the first definition of the true rake angle of a turning tool, and reported that orthogonal metal cutting occurs when the inclination is zero. However, Stabler [6] did not accept this definition of the angle because the chip flow direction was not considered. He proposed an alternative concept of the effective rake angle to address this limitation. A number of researchers agreed with Stabler, who also considered the cutting process in the definition of the tool rake angle. In contrast, Galloway [7] proposed the concept of the maximum rake system, which enabled the rake face to be ground through direct adjustment of the assigned angles on the machine vice.

However, these rake angle definitions do not consider the tool installation and cutting process conditions. Thus, Osman and Muller [8] developed a method based on the transformation matrix between the tool grinding, machine tool and tool working reference systems. To avoid the complex calculations required to describe the rake angle in different coordinate systems [8], Shi [9] proposed a new method that converted the spatial geometry problem to a plane geometry problem to calculate the rake angle more easily. This method was further extended by [10], providing a straightforward plane geometry routine to analyze the rake angle in both tool-in-use and tool-in-hand systems.

The rake angles definitions in [7,9,10] are based only on the design, manufacture, or measurements of the tool without considering the cutting conditions. As cutting tools are designed to cut workpieces, some researchers [9,10,11] believe that the rake angle definitions in [5,7,8] should have reflected the cutting process. They proposed that the normal rake angle (NRA) of the tool should be used instead because the NRA directly affects the cutting power of the machine.

Grzesik [11] proposed a vector method to calculate the NRA in three-dimensional space. Hsieh [12] developed a kinematic model for NRA that considered the mutual relationships between the design and setting. Sambhav and Tandon et al. [13] established a forward and inverse mapping that related the grinding angle to the NRA.

To analyze the influence of the NRA on the cutting force, Armarego [14] derived analytical expressions for the dependence of the turning force on the NRA. In [15,16], the relationship between the cutting force and the NRA was investigated using finite element analysis. Later, Courbon and Fabre et.al [17] gave the contact pressure of element by FE. Gyunay and Korkut et al. [18,19] experimentally determined that as the NRA increases, the cutting force gradually decreases. Saglam and Yaldiz et al. [20] proposed that the NRA affects the full component of the cutting force during turning. Shih [21] simulated cuttings at four NRA values using the finite element method and found that the average and total cutting forces in all directions decrease with increasing NRA. The influence of the NRA on the cutting force also changed the machine tool power required in the machining process. Wu and Li et al. [22] reported a non-linear increase in the energy required for turning when the NRA exceeded a critical negative value. The chip shape is also dependent on the NRA. Menezes and Avdeev et al. [23] found that the NRA affects the formation process, morphology, and size of discontinuous chips. In [24,25], the chip forming conditions were obtained for the minimum NRA in a nano-cutting process.

However, in the aforementioned studies, it was assumed that the design rake angle was equal to the working normal rake angle (WNRA), and the variation in the cutting velocity direction at each point on the cutting edge was not considered. In fact, the NRA is closely related to the cutting velocity direction. Unlike traditional oblique cutting, in which the cutting velocity is generated by the movement of the tool [26], the cutting velocity of a turning tool is generated by rotation of the workpiece. The influence of the cutting velocity magnitude on the cutting force was studied in [27,28,29,30,31].

Based on analysis of the cutting velocity direction, a new model for calculating the WNRA and working inclination angle (WIA) of the active cutting edge (ACE) and the cutting force coefficient is proposed in this study. The cutting velocity direction at any point on the ACE is determined by the geometry of the tool and the radius of the workpiece. The WNRA and WIA of the cutting edge are accurately calculated based on the geometrical structure of the tool and workpiece. The cutting force coefficient is calculated using the WNRA and WIA. The main contribution of the proposed model is that the three-dimensional structure of the cutting edge and the continuous change in the cutting velocity direction are considered, along with the advantage that the WNRA and WIA at any point on the ACE can be accurately calculated for evaluation of the cutting force coefficient. The cutting force at the actual WNRA is predicted and compared with experimental results to demonstrate the effectiveness of the model.

## 2. Modeling of WNRA and WIA

Accurate calculation of the turning tool geometry angle is required for high-precision prediction of the turning force. Of the geometrical angles related to the turning tool, the WNRA an WIA directly affect the turning force of the tool. In traditional oblique cutting, the cutting force coefficients are known to be nonlinear function of the WNRA and WIA. In the turning force coefficient model in [26], the WNRA is also a function of the orthogonal rake angle and the WIA. To calculate the WNRA, the orthogonal rake angle must be first calculated, because the orthogonal rake angle varies along the ACE. A new direct analytical model for calculating the WNRA and WIA at any position on the cutting edge is proposed in this study. Variation in the cutting velocity direction along the ACE is considered in the model.

### 2.1. Introduction to Turning Tool Geometry

**Figure 1:**
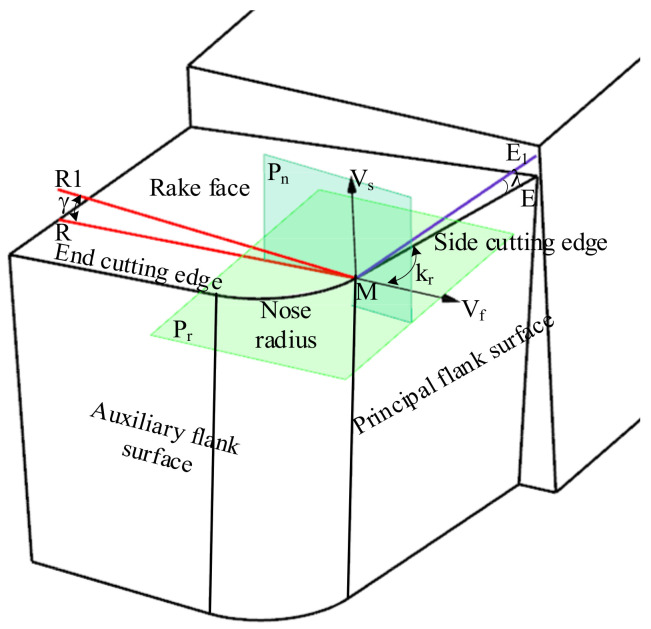
Definition of rake angle and inclination angle of turning tool.

The structure of the turning tool includes the rake face, major flank, minor flank, major cutting edge, minor cutting edge, corner, and shank, as shown in Figure 1. The turning tool angle is the basis for designing, manufacturing, grinding and measuring the tool.

The turning tool can be described by seven parameters $\gamma ,\;\lambda ,\;\alpha_{r},\;\alpha_{e},\;k_{r},\;k_{e}\;\mathrm{and}\;r$ as defined in ISO 3002-1 the NRA, inclination angle, side clearance angle, end clearance angle, side cutting edge angle, end cutting edge angle and tool nose radius, respectively. To describe different angles of the tool, a reference plane is defined, as shown in Figure 1. Point M is an arbitrary point on the ACE. *V*_s_ is the assumed turning velocity direction at point M and is referred to as the main direction of motion. *V*_f_ is the assumed feed direction at point M. At any point on the ACE, the direction of *V*_s_ and *V*_f_ are constant. *V*_s_ is the vector normal to the reference plane P_r_ through point M.

The normal plane P_n_ through point M is perpendicular to the cutting edge. The intersection line between plane P_n_ and plane P_r_ defines the line MR_1_, and the intersection line between the rake face and P_n_ defines the line MR. The angle between the lines MR and MR_1_ defines the rake angle γ. Projection of the main cutting edge ME onto the plane P_r_ defines the line ME_1_, and the angle between lines ME and ME_1_ defines the inclination angle λ, The inclination angle λ affects the flow direction of the chips. The angle between lines ME_1_ and *V*_f_ defines the side cutting edge angle *k*_r_. The side cutting edge angle *k*_r_ affects the length of the ACE. Obviously, reference plane P_r_ is determined by the cutting velocity direction *V*_s_. The orientation of MR_1_ is closely related to P_r_ and affects the WNRA and WIA.

### 2.2. Definition of ACE and Discretization of Cutting Edge 

The main cutting edge in the turning process includes the straight cutting edge and the tool nose. When the cutting depth is large, the cutting force comes mainly from the straight cutting edge. Relatively little material is removed by the tool nose, thus, the cutting force generated by the tool nose accounts for a small proportion of the total cutting force. In contrast, for material that is difficult to machine, such as nickel-based superalloys, the cutting force of the tool nose accounts for a significant proportion of the total cutting force due to the small amount of material cut, and only the corner of the tool participates in cutting. The cutting edge is curved on the tool nose. Thus, the representation of the cutting edge on the tool nose must be analyzed.

**Figure 2:**
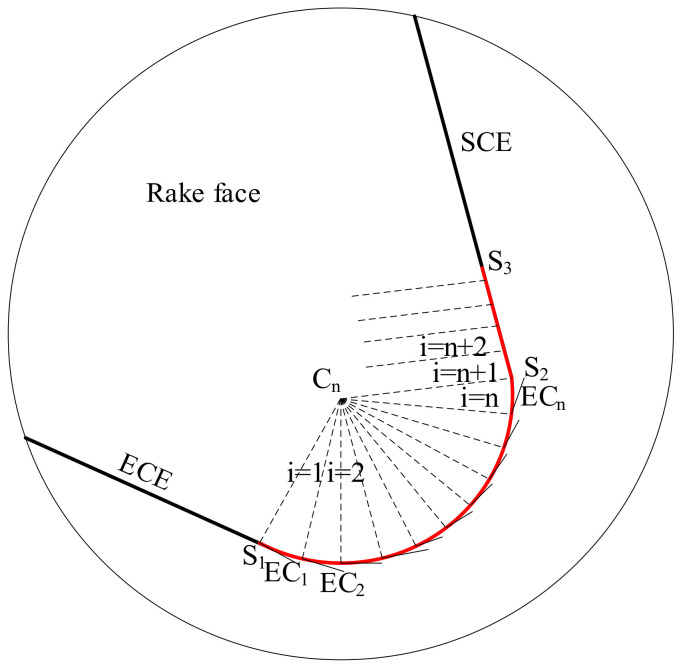
Diagrams of ACE and equivalent cutting edges.

In actual machining the ACE is the part of the turning tool cutting edge that engages with the workpiece. In Figure 2, the red curve S_1_S_3_ is the ACE and includes the curve S_1_S_2_ on the tool nose and the line S_2_S_3_ on the straight cutting edge. C_n_ is at the center of the tool nose corner. The ACE is divided into several elements of equal curve length. The straight line EC_1_ is the tangent of the ACE at point S_1_, and it represents the equivalent cutting edge of the first element S_1_.

### 2.3. Analysis of Change in Cutting Velocity Direction during Turning

**Figure 3:**
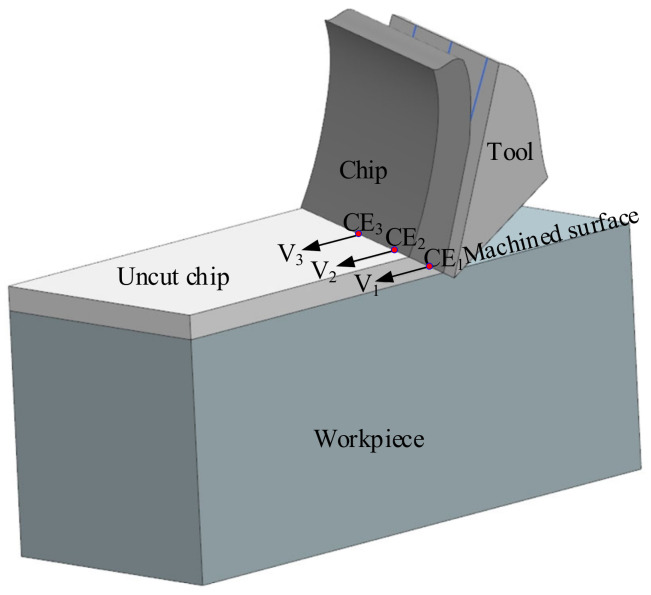
Diagram of velocity directions in oblique cutting.

In traditional oblique cutting, the workpiece is fixed, and the tool moves continuously along the cutting velocity direction, which is generated by the movement of the tool. At any point on the cutting edge, the cutting velocity direction is parallel to the motion of the tool, as shown in Figure 3. 

**Figure 4:**
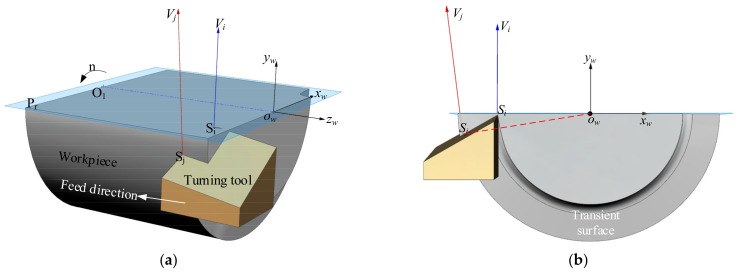
Actual cutting velocity direction along the cutting edge; (**a**) ISO view; (**b**) Z_w_ view.

Figure 4a shows a diagram of the cylindrical turning process. Plane P_r_ is a reference plane that passes through the workpiece axis. The workpiece coordinate system x_w_-y_w_-z_w_ is established at the center o_w_ of the end face of the machined part. The positive z_w_ axis direction is opposite to the feed direction, the positive y_w_ axis direction is vertical upward, and x_w_ is obtained from the y_w_ and z_w_ axes, according to the right-hand rule cross product.

The cutting velocity is affected by the radius of the workpiece during turning, as shown in Figure 4a. To facilitate display and observation, the workpiece is sectioned, the arrow near rotation speed *n* indicates the rotation direction of the workpiece. At points S_i_ and S_j_ on the ACE, where the reference plane of point S_i_ is P_r_, the rotation of the workpiece produces velocities *V*_i_ and *V*_j_, respectively. Figure 4b shows that the cutting velocity *V*_i_ is parallel to the y_w_ axis at S_i_. The cutting velocity direction is not parallel to the y_w_ axis at other points on the ACE.

During the cutting process, the WNRA and WIA of the tool are defined in terms of the reference plane, and the normal vector of the reference plane at any point on the ACE is the cutting velocity direction at that point. Thus, variation in the cutting velocity direction along the points on the ACE can also change the WNRA and WIA.

### 2.4. Modeling of WNRA and WIA on ACE

**Figure 5:**
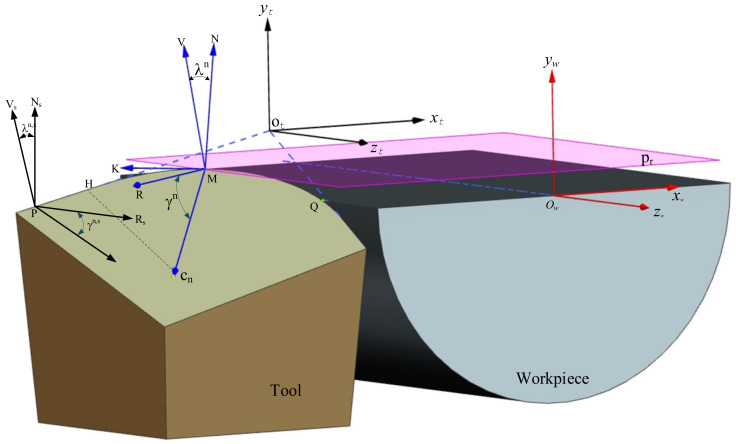
Illustration of the working normal rake angle (NRA) and inclination angle on arc.

An arbitrary point M on the ACE is selected, as shown in Figure 5. The intersection of the extension of side cutting edge and end cutting edge serves as the origin of the tool coordinate system x_t_-z_t_-y_t_, in which the x_t_, y_t_, and z_t_ axes are parallel to the x_w_,y_w_, and z_w_ axes.

Following the method described in [32], the coordinates of any point M on the ACE in the tool coordinate system can be expressed as

(1) $$C_{M}^{t}\left(\theta \right)=\left[\begin{array}{c} x_{{}_{M}}^{t}\left(\theta \right)\\ y_{M}^{t}\left(\theta \right)\\ z_{M}^{t}\left(\theta \right)\\ 1 \end{array}\right]=T_{4\times 4}\times R_{4\times 4}^{z}\times R_{4\times 4}^{x}\times \left[\begin{array}{c} r\cdot \cos\theta \\ 0\\ -r\cdot \sin\theta \\ 1 \end{array}\right]$$

where the transformation matrices $T_{4\times 4},R_{4\times 4}^{z},R_{4\times 4}^{x}$ are calculated according to Trans, Rot_Zt_, and Rot_Xt_ respectively in Equation (7) in [32]. In the workpiece coordinate system, the vector **O**_w_**O**_t_ is expressed as ${\left[x_{o_{w}o_{t}},y_{o_{w}o_{t}},z_{o_{w}o_{t}}\right]}^{T}$, and calculated according to Equation (22) in [32], the coordinates of point M in the workpiece coordinate system can be calculated as

(2) $$C_{M}^{w}\left(\theta \right)=\left[\begin{array}{c} x_{{}_{M}}^{w}\left(\theta \right)\\ y_{{}_{M}}^{w}\left(\theta \right)\\ z_{{}_{M}}^{w}\left(\theta \right)\\ 1 \end{array}\right]=M_{4\times 4}\times C_{M}^{t}\left(\theta \right)$$

where the homogeneous transformation matrix **M**_4×4_ is calculated according to Equation (26) in [32]. The intersection between the normal plane $p_{n}^{M}$ of point M and the rake face is the vector **MC**_n_. As defined in Section 2.2, the equivalent cutting edge at point M given by the vectors **MK**, **MK** and **MC**_n_ in the workpiece coordinate system can be calculated as

(3) $$MC_{n}^{t}\left(\theta \right)=\left[\begin{array}{c} x_{{}_{MC}}^{{}_{t}}\left(\theta \right)\\ y_{{}_{MC}}^{{}_{t}}\left(\theta \right)\\ z_{{}_{MC}}^{{}_{t}}\left(\theta \right)\\ 0 \end{array}\right]=R_{4\times 4}^{z}\times R_{4\times 4}^{x}\times \left[\begin{array}{c} -r\cdot \cos\theta \\ 0\\ r\cdot \sin\theta \\ 0 \end{array}\right]$$

Because $MC_{n}^{t}=MC_{n}^{w}$, the vector **MK** can be calculated as

(4) $$MK^{t}\left(\theta \right)=\left[\begin{array}{c} x_{{}_{MK}}^{{}_{t}}\left(\theta \right)\\ y_{{}_{MK}}^{{}_{t}}\left(\theta \right)\\ z_{{}_{MK}}^{{}_{t}}\left(\theta \right)\\ 0 \end{array}\right]=R_{4\times 4}^{z}\times R_{4\times 4}^{x}\times \left[\begin{array}{c} -r\cdot \sin\theta \\ 0\\ -r\cdot \cos\theta \\ 0 \end{array}\right]$$

The cutting velocity direction at point M is the vector **MV**, derived as

(5) $$MV^{w}\left(\theta \right)={\left[-y_{{}_{M}}^{w}\left(\theta \right),x_{{}_{M}}^{w}\left(\theta \right),0,0\right]}^{T}$$

The angle between **MV** and **y**_w_ can be expressed as

(6) $$\rho_{{}_{M}}^{w}\left(\theta \right)=\arccos\left(\frac{MV^{w}\left(\theta \right)\cdot y_{w}}{\left|MV^{w}\left(\theta \right)\right|}\right)$$

where $\left|MV^{w}\left(\theta \right)\right|=\sqrt{{\left[-y_{{}_{M}}^{w}\left(\theta \right)\right]}^{2}+{\left[x_{{}_{M}}^{w}\left(\theta \right)\right]}^{2}}$. The intersection line of planes $P_{n}^{M}$ and $P_{r}^{M}$ is the vector **MR**, which can be calculated as

(7) $$MR^{{}^{w}}\left(\theta \right)=MV^{w}\left(\theta \right)\times MK^{w}\left(\theta \right)=\left[\begin{array}{c} x_{{}_{MV}}^{w}\left(\theta \right).z_{{}_{MK}}^{w}\left(\theta \right)\\ y_{{}_{MV}}^{w}\left(\theta \right).z_{{}_{MK}}^{w}\left(\theta \right)\\ -y_{{}_{MV}}^{w}\left(\theta \right).y_{{}_{MK}}^{w}\left(\theta \right)-x_{{}_{MK}}^{w}\left(\theta \right).x_{{}_{MV}}^{w}\left(\theta \right)\\ 0 \end{array}\right]$$

Thus, the WNRA $\gamma_{M}^{n}\left(\theta \right)$ at point M can be obtained as

(8) $$\gamma_{{}_{M}}^{n}\left(\theta \right)=\mathrm{arc}\cos\left(\frac{MR^{w}\left(\theta \right).MC_{n}^{w}\left(\theta \right)}{\left|MR^{w}\left(\theta \right)\right|.\left|MC_{n}^{w}\left(\theta \right)\right|}\right)$$

where

(9) $$\begin{array}{l} \left|MR^{w}\left(\theta \right)\right|=\sqrt{{\left[x_{{}_{MV}}^{w}\left(\theta \right)\right]}^{2}+{\left[y_{{}_{MV}}^{w}\left(\theta \right)\cdot z_{{}_{MK}}^{w}\left(\theta \right)\right]}^{2}+{\left[y_{{}_{MV}}^{w}\left(\theta \right)\cdot y_{{}_{MK}}^{w}\left(\theta \right)+x_{{}_{MV}}^{w}\left(\theta \right)\right]}^{2}}\\ \left|MC_{{}_{n}}^{w}\left(\theta \right)\right|=\sqrt{{\left[x_{{}_{MC}}^{w}\left(\theta \right)\right]}^{2}+{\left[y_{{}_{MC}}^{w}\left(\theta \right)\right]}^{2}+{\left[z_{{}_{MC}}^{w}\left(\theta \right)\right]}^{2}} \end{array}$$

The acute angle formed by the cutting velocity direction and the vertical line of the cutting edge is the WIA. In the plane formed by the cutting velocity and the cutting edge element, the direction MN perpendicular to the cutting edge MK can be calculated as

(10) $$\begin{array}{cc} MN\left(\theta \right) & =MK^{w}\left(\theta \right)\times MR^{w}\left(\theta \right)\\ & =\left[\begin{array}{c} -y_{{}_{MK}}^{w}\left(\theta \right).\left[y_{{}_{MV}}^{w}\left(\theta \right).y_{{}_{MK}}^{w}\left(\theta \right)+x_{{}_{MK}}^{w}\left(\theta \right).x_{{}_{MV}}^{w}\left(\theta \right)\right]-z_{{}_{MK}}^{w}\left(\theta \right).y_{{}_{MV}}^{w}\left(\theta \right).z_{{}_{MK}}^{w}\left(\theta \right)\\ z_{{}_{MK}}^{w}\left(\theta \right).x_{{}_{MV}}^{w}\left(\theta \right).z_{{}_{MK}}^{w}\left(\theta \right)+x_{{}_{MK}}^{w}\left(\theta \right).\left[y_{{}_{MV}}^{w}\left(\theta \right).y_{{}_{MK}}^{w}\left(\theta \right)+x_{{}_{MK}}^{w}\left(\theta \right).x_{{}_{MV}}^{w}\left(\theta \right)\right]\\ x_{{}_{MK}}^{w}\left(\theta \right).y_{{}_{MV}}^{w}\left(\theta \right).z_{{}_{MK}}^{w}\left(\theta \right)-x_{{}_{MV}}^{w}\left(\theta \right).z_{{}_{MK}}^{w}\left(\theta \right).z_{{}_{MK}}^{w}\left(\theta \right)\\ 0 \end{array}\right] \end{array}$$

Because MN is perpendicular to the equivalent cutting edge MK, the WIA $\lambda_{M}^{n}$ at point M is equal to the angle formed by the cutting velocity MV and the vertical line MN, and is expressed as

(11) $$\lambda_{M}^{n}\left(\theta \right)=\mathrm{arc}\cos\left(\frac{MN^{w}\left(\theta \right).MV^{w}\left(\theta \right)}{\left|MN^{w}\left(\theta \right)\right|.\left|MV^{w}\left(\theta \right)\right|}\right)$$

where

(12) $$\begin{array}{l} \left|MN^{w}\right|=\sqrt{{\left(x_{{}_{MN}}^{w}\right)}^{2}+{\left(y_{{}_{MN}}^{w}\right)}^{2}+{\left(z_{{}_{MN}}^{w}\right)}^{2}}\\ \left|MV^{w}\right|=\sqrt{{\left(x_{{}_{MV}}^{w}\right)}^{2}+{\left(y_{{}_{MV}}^{w}\right)}^{2}} \end{array}$$

The projection of the equivalent cutting edge MK on the x_w_-y_w_ plane can be expressed as

(13) $$MK_{xy}^{w}\left(\theta \right)=\left[\begin{array}{c} x_{{}_{MK}}^{{}_{w}}\left(\theta \right)\\ y_{{}_{MK}}^{{}_{w}}\left(\theta \right)\\ 0\\ 0 \end{array}\right]$$

The working side cutting edge angle (WSCEA) at point M can be calculated as

(14) $$k_{r}\left(\theta \right)=\arccos\left(\frac{MK_{xy}^{w}\left(\theta \right)\cdot z_{w}}{\left|MK_{xy}^{w}\left(\theta \right)\right|}\right)$$

where the vector **z_w_** = [0,0,1,0]^T^, the ACE consists of a tool nose and a straight cutting edge. Owing to the continuous variation in the cutting velocity direction on the straight cutting edge, the WNRA and WIA on the straight cutting edge also change. 

The angle between the side cutting edge and the end cutting edge is ε.The unit vector **u**^t^ of the side cutting edge can be expressed as

(15) $$u^{t}=\left[\begin{array}{c} x_{u}^{t}\\ y_{u}^{t}\\ z_{u}^{t}\\ 1 \end{array}\right]=\left[\begin{array}{c} \cos\left(\lambda \right)\cdot \cos\left(k_{r}\right)\\ -\sin\left(\lambda \right)\\ -\cos\left(\lambda \right)\cdot \sin\left(k_{r}\right)\\ 1 \end{array}\right]$$

and the coordinate of point H can be obtained as

(16) $$x_{H}=\left|O_{t}H\right|\cdot x^{t}$$

where $\left|O_{t}H\right|=\cot(\varepsilon /2)\cdot r$.

On the straight cutting edge, the direction vector of the intersection of the normal plane and the rake face is ${HC}_{n}^{t}$, which can be calculated as

(17) $$HC_{n}^{t}={\left[x_{HC_{n}}^{t},y_{HC_{n}}^{t},z_{HC_{n}}^{t}\right]}^{T}$$

The coordinates of arbitrary point P on the straight cutting edge of the ACE can be determined as

(18) $$P^{t}=\left[\begin{array}{c} x_{p}^{t}\\ y_{p}^{t}\\ z_{p}^{t}\\ 1 \end{array}\right]=\left(\left|O_{t}H\right|+k\cdot dL\right)\cdot u^{t}\;\left(k=1,2\ldots ,\right)$$

which are written in the workpiece coordinate system as

(19) $$P^{w}=\left[\begin{array}{c} x_{p}^{w}\\ y_{p}^{w}\\ z_{p}^{w}\\ 1 \end{array}\right]=M_{4\times 4}\times P^{t}$$

The cutting velocity direction vector at point P is $PV^{t}={\left[-y_{p}^{w},x_{p}^{w},0\right]}^{T}$, and the direction vector PR_s_ of the intersection line between the normal plane and the rake face can be calculated as

(20) $$PR_{S}^{w}=PV_{S}^{w}\times u^{t}=\left[\begin{array}{c} x_{PR}^{w}\\ y_{PR}^{w}\\ z_{PR}^{w}\\ 1 \end{array}\right]=\left[\begin{array}{c} x_{p}^{w}\cdot z_{u}^{t}\\ y_{p}^{w}\cdot z_{u}^{t}\\ -y_{p}^{w}\cdot y_{u}^{t}-x_{p}^{w}\cdot x_{u}^{t}\\ 1 \end{array}\right]$$

The WNRA $\gamma_{p}^{n}$ at point P can be calculated as

(21) $$\gamma_{p}^{n}=\mathrm{arc}\cos\left(\frac{PR_{S}^{w}.HC_{n}^{w}}{\left|PR_{S}^{w}\right|.\left|HC_{n}^{w}\right|}\right)$$

where

(22) $$\begin{array}{l} \left|PR_{S}^{w}\right|=\sqrt{{\left(x_{PR}^{w}\right)}^{2}+{\left(y_{PR}^{w}\right)}^{2}+{\left(z_{PR}^{w}\right)}^{2}}\\ \left|HC_{n}^{w}\right|=\sqrt{{\left(x_{HC_{n}}^{w}\right)}^{2}+{\left(y_{HC_{n}}^{w}\right)}^{2}+{\left(z_{HC_{n}}^{w}\right)}^{2}} \end{array}$$

Similarly, the vector **PN** perpendicular to the cutting edge can be calculated as

(23) $$PN_{S}^{w}=u^{w}\times PR_{S}^{w}=\left[\begin{array}{c} x_{PN}^{w}\\ y_{PN}^{w}\\ z_{PN}^{w}\\ 1 \end{array}\right]=\left[\begin{array}{c} z_{u}^{w}\cdot y_{PR}^{w}+z_{PR}^{w}\cdot y_{u}^{w}\\ -x_{u}^{w}\cdot z_{PR}^{w}-x_{PR}^{w}\cdot z_{u}^{w}\\ x_{PR}^{w}\cdot y_{u}^{w}+y_{PR}^{w}\cdot x_{u}^{w}\\ 1 \end{array}\right]$$

The WIA at point P can be expressed as

(24) $$\lambda_{P}^{n}=\mathrm{arc}\cos\left(\frac{PN_{S}^{w}.PV_{S}^{w}}{\left|PN_{S}^{w}\right|.\left|PV_{S}^{w}\right|}\right)$$

where

(25) $$\begin{array}{l} \left|PN_{S}^{w}\right|=\sqrt{{\left(x_{PN}^{w}\right)}^{2}+{\left(y_{PN}^{w}\right)}^{2}+{\left(z_{PN}^{w}\right)}^{2}}\\ \left|PV_{S}^{w}\right|=\sqrt{{\left(y_{PV}^{w}\right)}^{2}+{\left(x_{PV}^{w}\right)}^{2}} \end{array}$$

**Figure 6:**
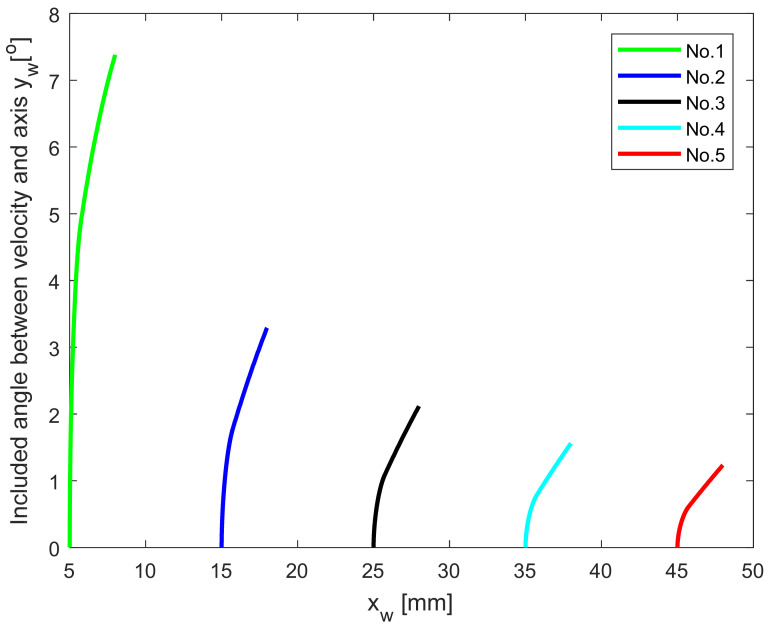
Included angle between velocity and y_w_ axis for different workpiece radii.

To understand the relationship between the cutting velocity direction on the ACE and the radius of the machined part, a sensitivity study was conducted by changing the radius of the workpiece. For the simulation, the NRA was set as $\gamma^{n}=24.7{}^{\circ}$, the inclination angle was $\lambda^{n}=13.6{}^{\circ}$, the side cutting edge angle was *k*_r_ = 93°, the end cutting edge angle is *k*_e_ = 52°, the tool nose radius is *r* = 0.8 mm and the feed rate is *f* = 0.1 mm. The radius of the workpiece is shown in Table 1. The angle between the velocity direction at each point on the ACE and the **y**_w_ axis was calculated to characterize the change in velocity direction based on Equation (6); the results are shown in Figure 6.

The cutting velocity direction is closely related to the radius of the workpiece, as shown in Figure 6. The largest range of the cutting velocity direction occurred in Test No.1, for which the maximum angle with the y_w_ axis was 7.4°. The minimum angle change of 1.2 ° occurred in Test No.5. As the workpiece radius increases, the range of the cutting velocity direction on the ACE gradually decreases. Thus, the radius of the workpiece exerts a size effect on the cutting velocity direction at each point on the ACE during turning.

**Figure 7:**
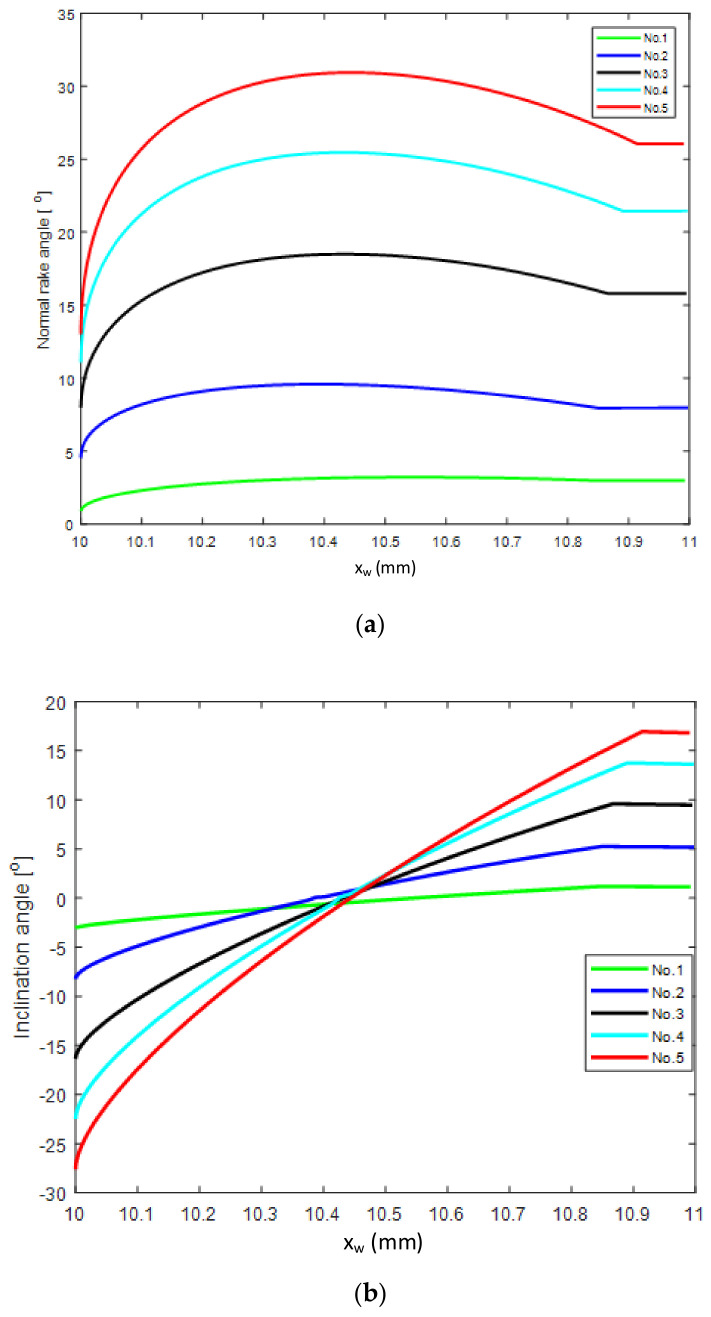

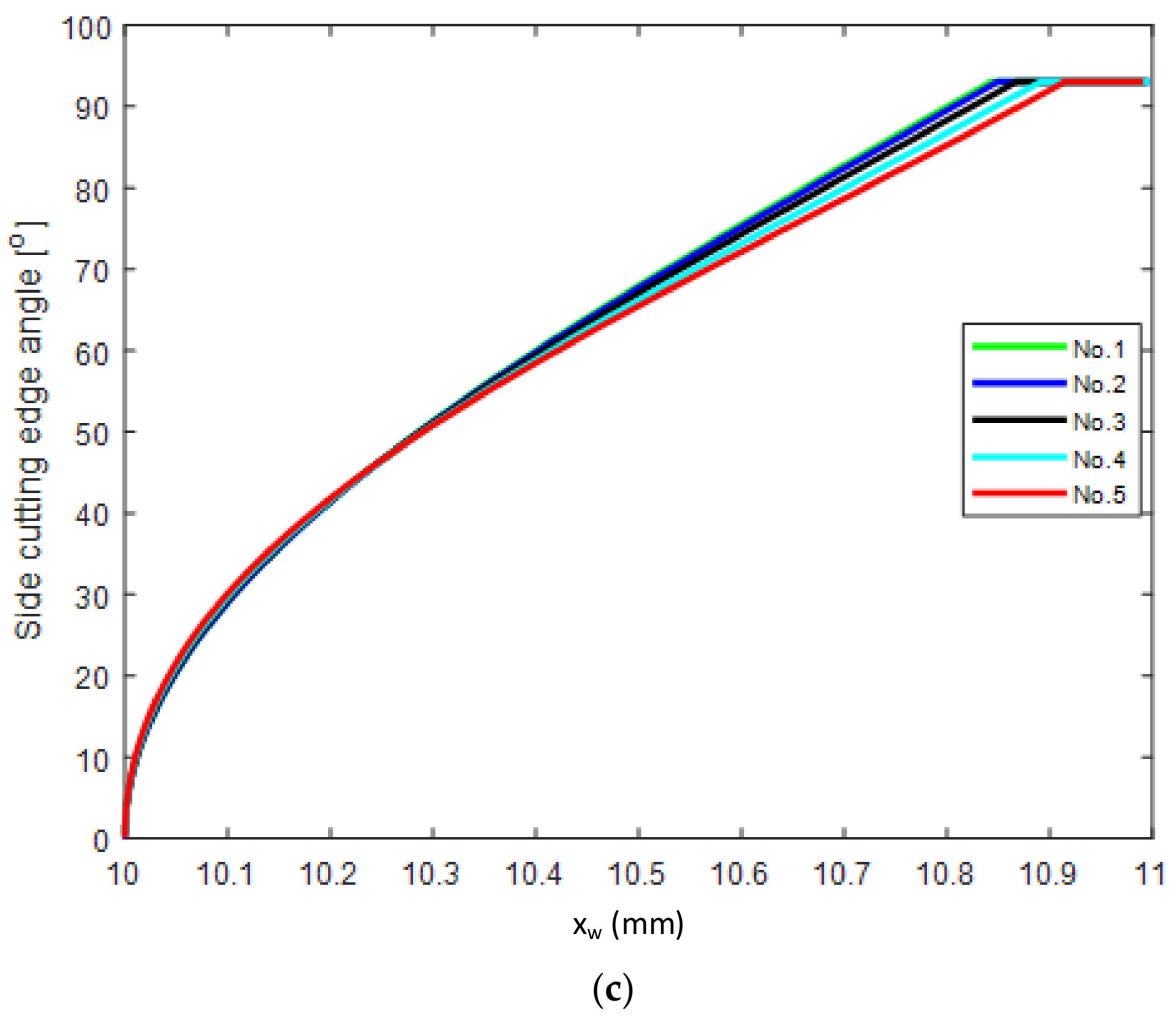
Actual angles along the cutting edge: (**a**) working normal rake angle (NRA); (**b**)working inclination angle; (**c**) side cutting edge angle on each elements.

To investigate the variation in the WNRA, WIA, and WSCEA with the design angle of the tool, the WNRA WIA and WSCEA were calculated at each element on the ACE for different NRA, inclination angle and side cutting edge angle values. The geometric parameters of the tool are shown in Table 2. The side cutting edge angle was set as 93°, the end cutting edge angle was 52°, the tool nose radius was *r* = 0.8 mm, the feed rate *f* = 0.1 mm, the cut depth a_p_ = 1 mm, and the workpiece radius *R*_w_ = 20 mm. The resulting WNRA $\gamma^{n}$, WIA $\lambda^{n}$ and WSCEA *k*_r_ for each element on the ACE are shown in Figure 7. The abscissa is the x_w_ coordinate of the element in the workpiece coordinate system.

Along the ACE from the tool nose to the straight cutting edge, the WNRA first increases and then decreases, as shown in Figure 7a. The variation range of the WNRA at each point on the tool nose is greater than the range on the straight edge of the ACE, where the WNRA values are nearly constant. Comparing the results from Test No. 1 and Test No. 5 in Table 2, it can be observed that the variation range of the WNRA for each element increases with the design rake angle. The WIA is affected by both the rake angle and the inclination angle (tool-in-hand system) as shown in Figure 7b. Starting from the tool nose, the WIA gradually increases along the curved cutting edge, but does not change significantly along the straight cutting edge. The variation range of the WIA is positively correlated with the design rake angle. In Figure 7b, all lines increase from negative to positive values. Thus, in the curved part of the ACE, there is always a point with an inclination angle of zero, at which orthogonal cutting occurs at the element cutting edge, whereas oblique cutting occurs at other points on the ACE.

Figure 7c shows the WSCEA along the ACE. The WSCEA gradually increases from the curved to the straight cutting edge and reaches its maximum value on the straight cutting edge. The maximum WSCEA value is equal to the design side cutting edge angle of the tool.

## 3. Turning Force Modeling Considering Actual WNRA and WIA along Cutting Edge

Accurate calculation of the turning force coefficient is important for high-precision prediction of the turning force. Reference [26] reported that the turning force coefficient is a function of the WNRA and WIA. In this section, the cutting force coefficient is calculated using the WNRA and WIA values accurately calculated in Section 2.4. An analytical calculation model of the uncut chip thickness (UCT) along the cutting edge of the element is also presented, and the turning force is predicted.

### 3.1. Mechanism of Turning Force Model

**Figure 8:**
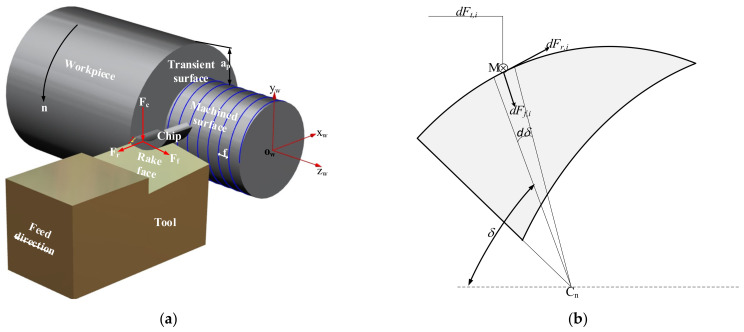
Diagram of turning process: (**a**) Turning forces on the tool; (**b**) The force components acting on the element.

The cutting force on the tool is shown in Figure 8a. The force is derived from the shear deformation force of the material in the shear plane. These forces are called the radial force *F*_r_, the cutting force *F*_t_, and the feed force *F*_f_. The cutting, feed and radial forces acting on the *i*^th^ cutting edge element are denoted as *dF_t,i_*, *dF_f,i_*, and *dF_r,i_*, respectively.

As shown in Figure 8b, the angular location of the *i*th element is δ, and the force components acting on the element can be expressed as

(26) $$\left[\begin{array}{c} dF_{t,i}\left(\delta \right)\\ dF_{f,i}\left(\delta \right)\\ dF_{r,i}\left(\delta \right) \end{array}\right]=\left[\begin{array}{cc} k_{tc,i}\left(\delta \right) & k_{tE}\\ k_{fc,i}\left(\delta \right) & k_{fE}\\ k_{rc,i}\left(\delta \right) & k_{rE} \end{array}\right]\left[\begin{array}{c} dA_{i}\left(\delta \right)\\ dL \end{array}\right]$$

where $k_{tc}\left(\delta \right),k_{fc}\left(\delta \right)$ and $k_{rc}\left(\delta \right)$ are the cutting force coefficients; *k_te_*, *k_fe_* and *k_re_* are the edge coefficients; $dA_{i}\left(\delta \right)$ and *dL* are the uncut chip area and length of the *i*th element, respectively.

### 3.2. Analysis of Cutting Force Coefficients

According to Ref. [26], the cutting force coefficients during oblique cutting can be expressed as shown in Equation (27). They are derived from the friction, normal pressure on the rake face, and the physical relationship between the cutting velocity, chip velocity, and shear velocity with the assumptions that the basic mechanisms of oblique and orthogonal cutting are the same in the normal plane.

(27) $$\begin{array}{l} K_{tc,i}=\frac{\tau_{S}\cdot \cos(\beta_{i}^{n}-\gamma_{i}^{n})+tan\lambda_{i}^{n}\cdot tan\eta_{i}^{n}\cdot \sin\beta_{i}^{n}}{\sin\varphi_{i}^{n}\cdot G\left(\varphi ,\beta ,\gamma ,\eta \right)}\\ K_{fc,i}=\frac{\tau_{S}\cdot Sin(\beta_{i}^{n}-\gamma_{i}^{n})}{\sin\varphi_{i}^{n}\cdot \cos\lambda_{i}^{n}\cdot G\left(\varphi ,\beta ,\gamma ,\eta \right)}\\ K_{rc,i}=\frac{\tau_{S}\cdot \left(\cos(\beta_{i}^{n}-\gamma_{i}^{n})\cdot tan\lambda_{i}^{n}+\tan\eta_{i}\cdot \sin\beta_{i}^{n}\right)}{\sin\varphi_{i}^{n}\cdot G\left(\varphi ,\beta ,\gamma ,\eta \right)} \end{array}$$

where $G\left(\varphi ,\beta ,\gamma ,\eta ,\theta \right)=\sqrt{\cos^{2}(\varphi_{i}^{n}+\beta_{i}^{n}-\gamma_{i}^{n})+tan^{2}\eta_{i}\cdot \sin^{2}\beta_{i}^{n}}$, and $\lambda_{i}^{n},\;\eta_{i}^{n},\;\varphi_{i}^{n},$ $\tau_{S},\;\beta_{i}^{n},\;\mathrm{and}\;\gamma_{i}^{n},$ are the WIA, chip flow angle, normal shear angle, shear yield stress, normal friction angle, and WNRA, respectively. The cutting force coefficient contains six unknown parameters. For a given machined part, the shear yield stress and geometric structure of the tool are known. Of the six parameters in Equation (27), the WIA $\lambda_{i}^{n}$ and WNRA $\gamma_{i}^{n}$ at the cutting edge of each element can be calculated using Equations (8), (11) and (24). Under the Stable’s chip flow criterion [26], the shear angle $\varphi_{i}^{n}$, average friction coefficient $\beta_{i}^{n}$, and shear yield stress $\tau_{S}$ with oblique cutting are the same as those under orthogonal machining conditions. Based on the Armarego and Stabler assumptions, three supplementary equations can be obtained

(28) $$\begin{array}{l} \varphi_{i}^{n}=\arctan\left(\frac{r_{c}\left(\cos\eta_{i}/\cos\lambda_{i}^{n}\right)\cdot \cos\gamma_{i}^{n}}{1-r_{c}\left(\cos\eta_{i}/\cos\lambda_{i}^{n}\right)\cdot \cos\gamma_{i}^{n}}\right)\\ \beta_{i}^{n}=\arctan(tan\beta_{a}.coS\eta_{i})\\ \eta_{i}^{n}=\lambda_{i}^{n} \end{array}$$

The cutting force coefficient of the element cutting edge can be obtained by substituting Equations (8), (11), (21), (24) and (28) into Equation (27).

**Figure 9:**
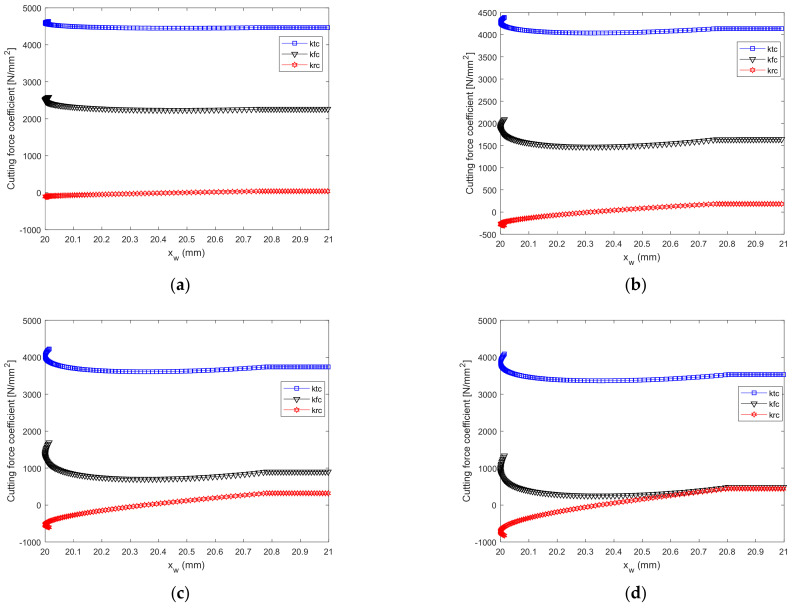

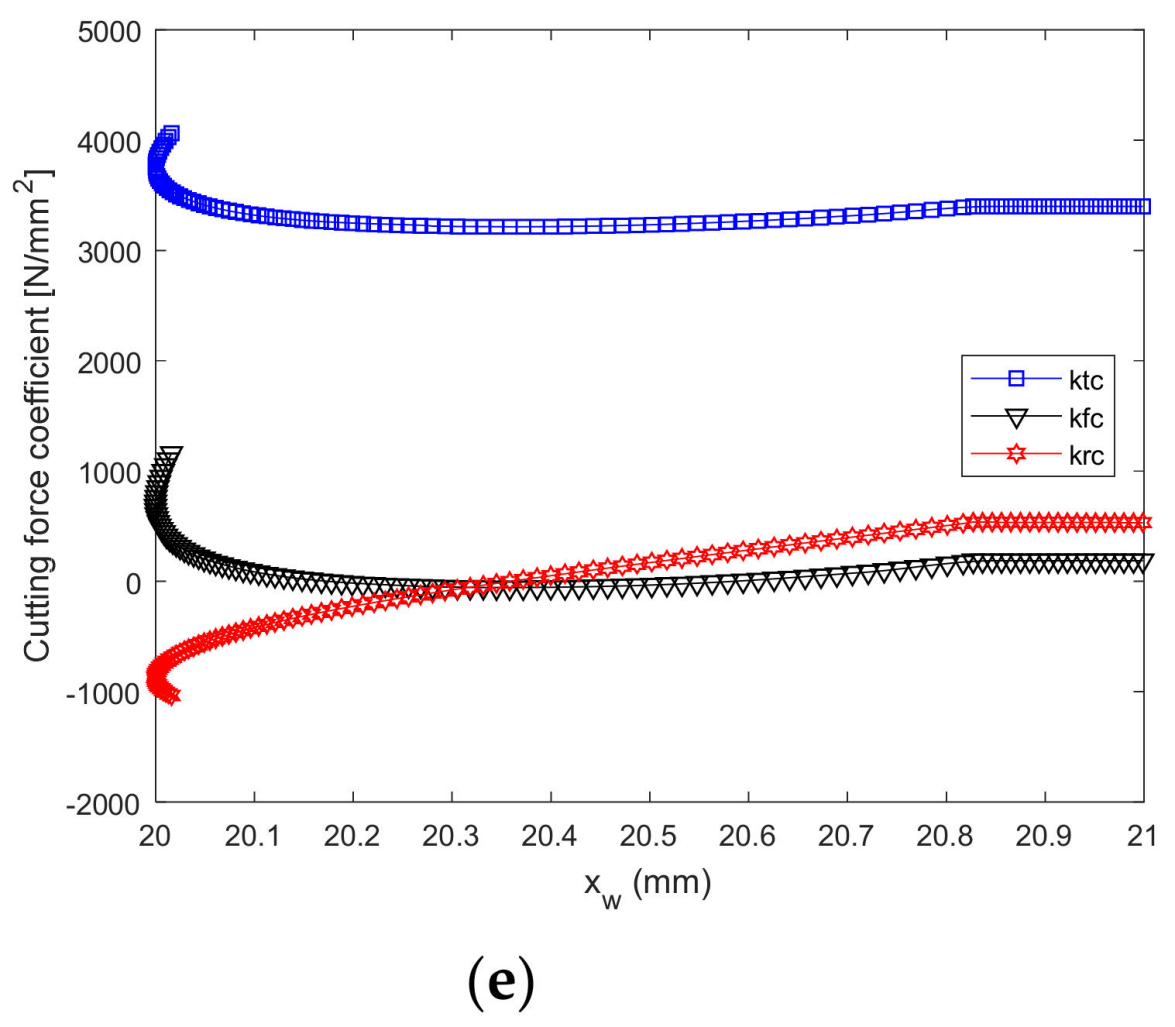
Cutting force coefficients for Test Nos. (**a**) 1; (**b**) 2; (**c**) 3; (**d**) 4; (**e**) 5.

The geometric parameters of the tool are presented in Table 2; the workpiece material used was GH4169, and the cutting force coefficient of each element was calculated along the ACE. Figure 9 shows the change in the cutting force coefficient with the tool geometry WNRA and WIA.

It is observed in Figure 9a–e that the tangential force coefficient *k_tc_* has the largest values, and the radial force coefficient *k_rc_* has the smallest value. *k_tc_*, *k_rc_*_,_ and *k_fc_* change significantly along the curved cutting edge of the ACE, but they change by only a small amount along the straight cutting edge. The cutting force coefficients in Figure 9a are the largest and those in Figure 9e are the smallest, mainly because the cutting force coefficient decreases with an increasing tool rake angle.

### 3.3. Modeling of UCT on Each Differential Element

**Figure 10:**
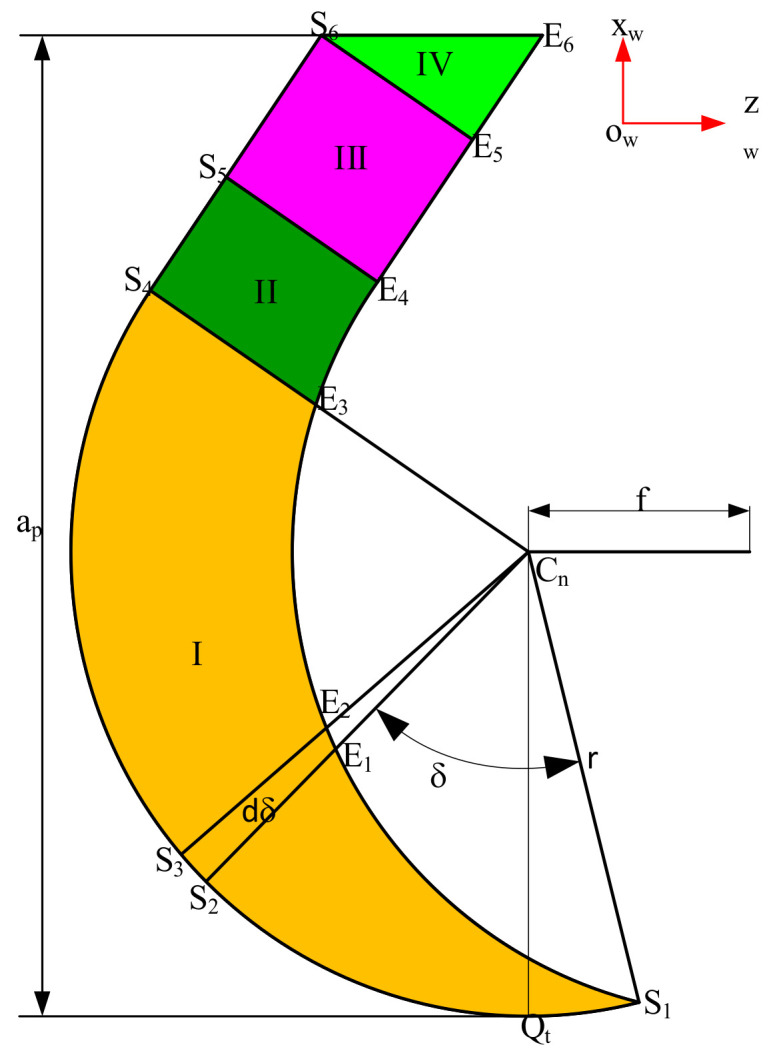
Undeformed chip thickness in cylindrical turning.

To calculate the differential element cutting force, the UCT must be calculated at the differential elements. The instantaneous position of the tool when it is completely engaged in the workpiece is known as the first tool position. After one cycle, the distance moved by the tool along the feed direction corresponds to the feed rate *f*, and the tool is in the second tool position. In Figure 10, points $S_{1},\;S_{2},\;S_{3},\;S_{4},\;S_{5},\;S_{6}\;\mathrm{and}\;Q_{t}$ are located at the second tool position, and points $S_{1},\;E_{1},\;E_{2},\;E_{3},\;E_{4},\;E_{5}\;\mathrm{and}\;E_{6}$ are located at the first tool position. $S_{1}$ is the intersection point of the tool curves at the first and second tool positions, and S_6_E_6_ is located on the surface to be machined. The UCT is divided into four zones labeled as I, II, III, and IV.

The angle (clockwise) between the line connecting an arbitrary element point on the ACE of the second tool position and the tool nose center C_n_, and the line segment $c_{n}S_{1}$ is denoted as the parameter angle δ. When a selected point on the ACE of the second tool position is shared by two adjacent areas, such as the point $S_{4},\;S_{5},\;S_{6}$, the angular location of the boundary point between zones I and II is denoted as $\delta_{1}$

(29) $$\delta_{1}=\frac{\pi }{2}-\mathrm{arc}\cos\left(\frac{f}{2r}\right)+k_{r}$$

The angular location of the boundary between zone II and III is denoted as $\delta_{2}$

(30) $$\delta_{2}=\frac{\pi }{2}-\mathrm{arc}\cos\left(\frac{f}{2r}\right)+k_{r}+\arctan\left(\frac{-\left(\frac{r}{\sin k_{r}}+f\right)\cos\left(k_{r}\right)-r\cdot \cot\left(\pi -k_{r}\right)}{r}\right)$$

The angular location of the boundary between zone III and IV is denoted as $\delta_{3}$

(31) $$\delta_{3}=\frac{\pi }{2}-\mathrm{arc}\cos\left(\frac{f}{2r}\right)+k_{r}+\arctan\left(\frac{\left(a_{p}-r\right)}{\cos\left(k_{r}-\frac{\pi }{2}\right)\cdot r}+\cot\left(k_{r}\right)\right)$$

The UCT *h_q_*, undeformed chip area *d*S*_q_* and undeformed chip length *dL_q_* (*q* = I, II, III, IV) at each differential element on the ACE are calculated as 

In zone I

(32) $$\begin{array}{l} h_{I\;}\left(\delta \right)=r-f\cdot \cos\left(\mathrm{arc}\cos\left(\frac{f}{2r}\right)+\delta \right)-\sqrt{r^{2}-f^{2}\cdot \sin^{2}\left(\mathrm{arc}\cos\left(\frac{f}{2r}\right)+\delta \right)}\\ dS_{I}\left(\delta \right)=\frac{r^{2}}{2}d\delta -\frac{1}{2}\left(f\cdot \cos\left(\mathrm{arc}\cos\left(\frac{f}{2r}\right)+\delta \right)+\sqrt{r^{2}-f^{2}\cdot \sin^{2}\left(\mathrm{arc}\cos\left(\frac{f}{2r}\right)+\delta \right)}\right)\cdot \\ \left(f\cdot \cos\left(\mathrm{arc}\cos\left(\frac{f}{2r}\right)+\delta +d\delta \right)+\sqrt{r^{2}-f^{2}\cdot \sin^{2}\left(\mathrm{arc}\cos\left(\frac{f}{2r}\right)+\delta +d\delta \right)}\right)\cdot \sin\left(d\delta \right)\\ dL_{I}=r\cdot d\delta \;\delta \in \left[0,\delta_{1}\right] \end{array}$$

In zone II

(33) $$\begin{array}{l} h_{II}\left(\delta \right)=-\left(-r\cdot \cot\left(k_{r}\right)+r\cdot \tan\left(\delta -\delta_{1}\right)\right)\cdot \tan\left(k_{r}\right)+\left(r+f\cdot \sin\left(k_{r}\right)+\left(-r\cdot \cot\left(k_{r}\right)+r\cdot \tan\left(\delta -\delta_{1}\right)\right)\cdot \tan\left(k_{r}\right)\right)\\ \;-\sqrt{r^{2}-{\left(\frac{r}{\sin\left(k_{r}\right)}+f+\frac{-r\cdot \cot\left(k_{r}\right)+r\cdot \tan\left(\delta -\delta_{1}\right)}{\cos\left(k_{r}\right)}\right)}^{2}\cdot \cos^{2}\left(k_{r}\right)}\\ dL_{II}\left(\delta \right)=r\cdot \tan\left(\delta -\delta_{1}\right),\\ dS_{II}\left(\delta \right)=h_{II}\cdot r\cdot \tan\left(\delta -\delta_{1}\right),\\ S_{II}=\int_{\delta_{1}}^{\delta_{2}}h_{II}\cdot d\left(r\cdot \tan\left(\delta -\delta_{1}\right)\right)\;\delta \in \left[\delta_{1},\delta_{2}\right] \end{array}$$

In zone III

(34) $$\begin{array}{l} h_{III}=f\cdot \sin\left(k_{r}\right)\\ L_{III}=\left(\frac{r}{\sin k_{r}}+f\right)\cdot \cos\left(k_{r}\right)+\frac{\left(a_{p}-r\right)}{\sin\left(k_{r}\right)}\\ S_{III}=f\cdot \sin\left(k_{r}\right)\cdot \left(\frac{\left(a_{p}-r\right)}{\sin\left(k_{r}\right)}+\left(\frac{r}{\sin\left(k_{r}\right)}+f\right)\cos\left(k_{r}\right)\right) \end{array}$$

In zone IV

(35) $$\begin{array}{l} h_{\mathrm{IV}}=t\cdot f\sin\left(k_{r}\right),\;t\in \left[0,1\right]\\ S_{\mathrm{IV}}=-\frac{1}{2}f^{2}\cdot \sin\left(k_{r}\right)\cdot \cos\left(k_{r}\right) \end{array}$$

**Figure 11:**
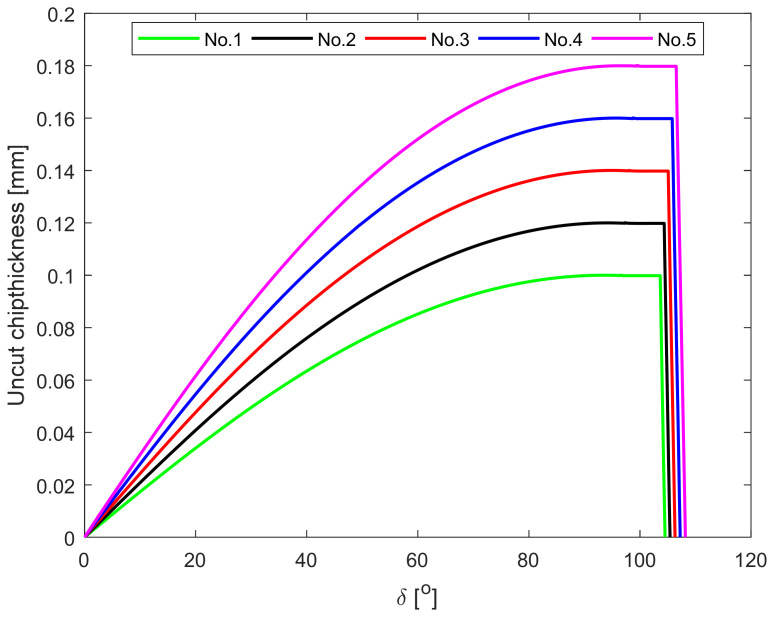
Uncut chip thickness on each element.

To analyze the variation in the UCT along each element of the ACE with the geometric angle of the tool and feed rate *f*, a sensitivity study was conducted using the geometric parameters of tools listed in Table 2, and the feed rates *f =* (0.1, 0.12, 0.14, 0.16, and 0.18) mm. Base on Equations (32)–(35), the change in UCT on each element along the ACE is shown in Figure 11.

As shown in Figure 11, on the tool nose curve, the UCT of the differential elements increases nonlinearly, and on the straight cutting edge, the UCT of the differential elements is constant. Comparing the results of the five tests in Figure 11, the UCT was found to increases with the feed rate. The end of each curve decreases linearly to zero because in zone IV, the expression for the UCT is a linear function.

From the analytical expression illustrated in Equations (32)–(35), it can be observed that the contact geometric relationship between the workpiece and the tool in the cutting process is a nonlinear function of (*r*, *k*_r,_ *f*, and *a*_p_). Because dynamometer in an experiment can only measure the force along the $x_{w},y_{w},z_{w}$ axes during turning, all differential element forces must be projected to the $x_{w},y_{w},z_{w}$ directions. In zone I, the projections of the element forces to the $x_{w},y_{w},z_{w}$ directions can be obtained as

(36) $$\begin{array}{l} dF_{x_{w}}^{I}\left(\delta \right)=\left[\begin{array}{cc} k_{fc,i}\left(\delta \right) & k_{\mathrm{fe}} \end{array}\right]\left[\begin{array}{c} dS_{I}\left(\delta \right)\cdot \cos\left(\delta \right)\\ dL_{I}\cdot \cos\left(\delta \right) \end{array}\right]-\left[\begin{array}{cc} k_{rc,i}\left(\delta \right) & k_{\mathrm{re}} \end{array}\right]\left[\begin{array}{c} dS_{I}\left(\delta \right)\cdot \sin\left(\delta \right)\\ dL_{I}\cdot \sin\left(\delta \right) \end{array}\right]\\ dF_{z_{w}}^{I}\left(\delta \right)=\left[\begin{array}{cc} k_{rc,i}\left(\delta \right) & k_{rE} \end{array}\right]\left[\begin{array}{c} dS_{I}\left(\delta \right)\cdot \cos\left(\delta \right)\\ dL_{I}\cdot \cos\left(\delta \right) \end{array}\right]+\left[\begin{array}{cc} k_{fc,i}\left(\delta \right) & k_{\mathrm{fe}} \end{array}\right]\left[\begin{array}{c} dS_{I}\left(\delta \right)\cdot \sin\left(\delta \right)\\ dL_{I}\cdot \sin\left(\delta \right) \end{array}\right]\\ dF_{y_{w}}^{I}\left(\delta \right)=\left[\begin{array}{cc} k_{tc,i}\left(\delta \right) & k_{tE} \end{array}\right]\left[\begin{array}{c} dS_{I}\left(\delta \right)\\ dL_{I} \end{array}\right] \end{array}$$

The total forces in zone I can be calculated as

(37) $$\begin{array}{l} F_{x_{w}}^{I}=\sum_{i=1}^{Y}\left(dF_{x_{w}}^{I}\left(\delta \right)\right)\\ F_{z_{w}}^{I}=\sum_{i=1}^{Y}\left(dF_{z_{w}}^{I}\left(\delta \right)\right)\;\delta \in \left[0,\delta_{1}\right]\\ F_{y_{w}}^{I}=\sum_{i=1}^{Y}\left(dF_{y_{w}}^{I}\left(\delta \right)\right) \end{array}$$

The total force projected from a differential element in zones II and III to the $x_{w},y_{w},z_{w}$ directions are

(38) $$\begin{array}{l} F_{x_{w}}^{II,III}=\sum_{j=1}^{N}\left(\left[\begin{array}{cc} k_{rc,i}\left(\delta \right) & k_{rE} \end{array}\right]\left[\begin{array}{c} \left(dS_{\mathrm{II}}+dS_{\mathrm{III}}\right)\cdot \sin\left(k_{r}\right)\\ \left(dL_{\mathrm{II}}+dL_{\mathrm{III}}\right)\cdot \sin\left(k_{r}\right) \end{array}\right]+\left[\begin{array}{cc} k_{fc,i}\left(\delta \right) & k_{fE} \end{array}\right]\left[\begin{array}{c} \left(dS_{\mathrm{II}}+dS_{\mathrm{III}}\right)\cdot \cos\left(k_{r}\right)\\ \left(dL_{\mathrm{II}}+dL_{\mathrm{III}}\right)\cdot \cos\left(k_{r}\right) \end{array}\right]\right)\\ F_{z_{w}}^{II,III}=\sum_{j=1}^{N}\left(\left[\begin{array}{cc} k_{rc,i}\left(\delta \right) & k_{rE} \end{array}\right]\left[\begin{array}{c} -\left(dS_{\mathrm{II}}+dS_{\mathrm{III}}\right)\cdot \cos\left(k_{r}\right)\\ -\left(dL_{\mathrm{II}}+dL_{\mathrm{III}}\right)\cdot \cos\left(k_{r}\right) \end{array}\right]+\left[\begin{array}{cc} k_{fc,i}\left(\delta \right) & k_{fE} \end{array}\right]\left[\begin{array}{c} \left(dS_{\mathrm{II}}+dS_{\mathrm{III}}\right)\cdot \sin\left(k_{r}\right)\\ \left(dL_{\mathrm{II}}+dL_{\mathrm{III}}\right)\cdot \sin\left(k_{r}\right) \end{array}\right]\right)\\ F_{y_{w}}^{II,III}=\sum_{j=1}^{N}\left(\left[\begin{array}{cc} k_{tc,i}\left(\delta \right) & k_{tE} \end{array}\right]\left[\begin{array}{c} \left(dS_{\mathrm{II}}+dS_{\mathrm{III}}\right)\\ \left(dL_{\mathrm{II}}+dL_{\mathrm{III}}\right) \end{array}\right]\right)\;\delta \in \left[\delta_{1},\delta_{2}\right] \end{array}$$

The total forces projected from a differential element in zone IV to the $x_{w},y_{w},z_{w}$ directions are

(39) $$\begin{array}{l} F_{x_{w}}^{\mathrm{IV}}=\left[\begin{array}{cc} k_{rc} & k_{fc} \end{array}\right]\left[\begin{array}{c} S_{\mathrm{IV}}\cdot \sin\left(k_{r}\right)\\ S_{\mathrm{IV}}\cdot \cos\left(k_{r}\right) \end{array}\right]\\ F_{z_{w}}^{\mathrm{IV}}=\left[\begin{array}{cc} -k_{rc} & k_{fc} \end{array}\right]\left[\begin{array}{c} S_{\mathrm{IV}}\cdot \cos\left(k_{r}\right)\\ S_{\mathrm{IV}}\cdot \sin\left(k_{r}\right) \end{array}\right]\\ F_{y_{w}}^{\mathrm{IV}}=k_{tc}\cdot S_{\mathrm{IV}} \end{array}$$

In zone IV, because the cutting edge is not in contact with the workpiece, there is no edge force. The total cutting force on the rake face projected to the $x_{w},y_{w},z_{w}$ directions is

(40) $$F_{q}=F_{q}^{I}+F_{q}^{\mathrm{II},III}+F_{q}^{\mathrm{IV}}\;q=x_{w},y_{w},z_{w}$$

The left term in the above expression can be measured directly during cutting experiments. The cutting force direction is measured using the dynamometer in a coordinate system which is different from the workpiece coordinate system. The cutting force measured by the dynamometer can be converted to the workpiece coordinate system as

(41) $$F_{q}^{w}=R_{k}F_{q}\;q=x_{w},y_{w},z_{w}$$

where $R_{k}=\left[\begin{array}{cccc} 0 & -1 & 0 & 0\\ 0 & 0 & -1 & 0\\ 1 & 0 & 0 & 0\\ 0 & 0 & 0 & 1 \end{array}\right]$.

The resultant force can be expressed as

(42) $$F^{w}=\sqrt{\sum {\left(F_{q}^{w}\right)}^{2}}\;q=x_{w},y_{w},z_{w}$$

### 3.4. Calculation of Edge Force Coefficients

After calculating the cutting force coefficient using Equations (26)–(28), the edge force coefficient must be calculated before the cutting force in the turning process can be predicted. In Equation (26), when the UCT is zero, there is only edge force in the turning process. Thus, through experimental measurement of the turning forces $F_{x},\;F_{y},\;\mathrm{and}\;F_{z}$, the linear function relating the turning force to the feed rate *f* is obtained, and the edge force coefficient is determined by linear fitting.

When the UCT is zero, it can be observed from Equations (37)–(39) that when $0\leq \delta \leq \delta_{1}$

(43) $$\begin{array}{l} F_{x_{w}}^{I}\left(\delta \right)=\left[\begin{array}{cc} k_{fE} & k_{rE} \end{array}\right]\left[\begin{array}{c} \sum_{i=1}^{n}\left(dL_{I}\cdot \cos\left(\delta \right)\right)\\ -\sum_{i=1}^{n}\left(dL_{I}\cdot \sin\left(\delta \right)\right) \end{array}\right]\\ F_{z_{w}}^{I}\left(\delta \right)=\left[\begin{array}{cc} k_{fE} & k_{rE} \end{array}\right]\left[\begin{array}{c} \sum_{i=1}^{n}\left(dL_{I}\cdot \sin\left(\delta \right)\right)\\ \sum_{i=1}^{n}\left(dL_{I}\cdot \cos\left(\delta \right)\right) \end{array}\right]\\ F_{y_{w}}^{I}\left(\delta \right)=k_{tE}\cdot \sum_{i=1}^{n}dL_{I} \end{array}$$

and when $\delta_{1}<\delta \leq \delta_{3}$

(44) $$\begin{array}{l} F_{x_{w}}^{II,III}=\sum_{j=1}^{N}\left(\left[\begin{array}{cc} k_{fE} & k_{rE} \end{array}\right]\left[\begin{array}{c} \left(dL_{\mathrm{II}}+dL_{\mathrm{III}}\right)\cdot \cos\left(k_{r}\right)\\ \left(dL_{\mathrm{II}}+dL_{\mathrm{III}}\right)\cdot \sin\left(k_{r}\right) \end{array}\right]\right)\\ F_{z_{w}}^{II,III}=\sum_{j=1}^{N}\left(\left[\begin{array}{cc} k_{fE} & k_{rE} \end{array}\right]\left[\begin{array}{c} \left(dL_{\mathrm{II}}+dL_{\mathrm{III}}\right)\cdot \sin\left(k_{r}\right)\\ -\left(dL_{\mathrm{II}}+dL_{\mathrm{III}}\right)\cdot \cos\left(k_{r}\right) \end{array}\right]\right)\;\delta \in \left[\delta_{1},\delta_{2}\right]\\ F_{y_{w}}^{II,III}=\sum_{j=1}^{N}\left(k_{tE}\cdot \left(dL_{\mathrm{II}}+dL_{\mathrm{III}}\right)\right)\; \end{array}$$

The total forces can be obtained as

(45) $$\left[\begin{array}{c} F_{x_{w}}\\ F_{z_{w}}\\ F_{y_{w}} \end{array}\right]=T\left[\begin{array}{c} k_{fE}\\ k_{rE}\\ k_{tE} \end{array}\right]$$

where

$T=\left[\begin{array}{ccc} \sum_{i=1}^{n}\left(dL_{I}\cdot \cos\left(\delta \right)\right)+\left(dL_{\mathrm{II}}+dL_{\mathrm{III}}\right)\cdot \cos\left(k_{r}\right) & -\sum_{i=1}^{n}dL_{I}\cdot \sin\left(\delta \right)+\left(dL_{\mathrm{II}}+dL_{\mathrm{III}}\right)\cdot \sin\left(k_{r}\right) & 0\\ \sum_{i=1}^{n}\left(dL_{I}\cdot \sin\left(\delta \right)\right)+\left(dL_{\mathrm{II}}+dL_{\mathrm{III}}\right)\cdot \sin\left(k_{r}\right) & dL_{I}\cdot \cos\left(\delta \right)-\left(dL_{\mathrm{II}}+dL_{\mathrm{III}}\right)\cdot \cos\left(k_{r}\right) & 0\\ 0 & 0 & \sum_{i=1}^{n}\left(dL_{I}\right)+dL_{\mathrm{II}}+dL_{\mathrm{III}} \end{array}\right]$ The edge force coefficient can be calculated as

(46) $$\left[\begin{array}{c} k_{fE}\\ k_{rE}\\ k_{tE} \end{array}\right]={\left[T\right]}^{-1}F_{e}$$

where the edge force is

$F_{e}=\left[\begin{array}{c} F_{x_{w}}\\ F_{z_{w}}\\ F_{y_{w}} \end{array}\right]$

## 4. Model Validation and Discussion

### 4.1. Workpiece Material

The turning force model established in Section 3 was verified through a series of turning experiments. The processed workpiece in the experiment was a round bar with a diameter of 80 mm and a length of 400 mm formed from the GH4169(LANZHU Co. Ltd., Shanghai, China). The hardness of GH 4169 is 44 HRC. The GH 4169 is nickel-based superalloy, which is broadly used in high temperature, aviation parts with aggressive environments and nuclear reactor systems. The material composition and mechanical properties are presented in Table 3 and Table 4, respectively [33].

### 4.2. Tool Selection and Cutting Parameter Design

Two tools (ZCC.CT Co., Ltd., Zhuzhou, China) and two corresponding tool handles (ZCC.CT Co., Ltd., Zhuzhou, China) were selected. The tools and tool handles parameters are presented in Table 5. The cutting experiments were conducted ten times, and the turning force of each machining was measured. The cutting parameters used in the turning process are presented in Table 6, the cutting speed was 30 m/min.

**Figure 12:**
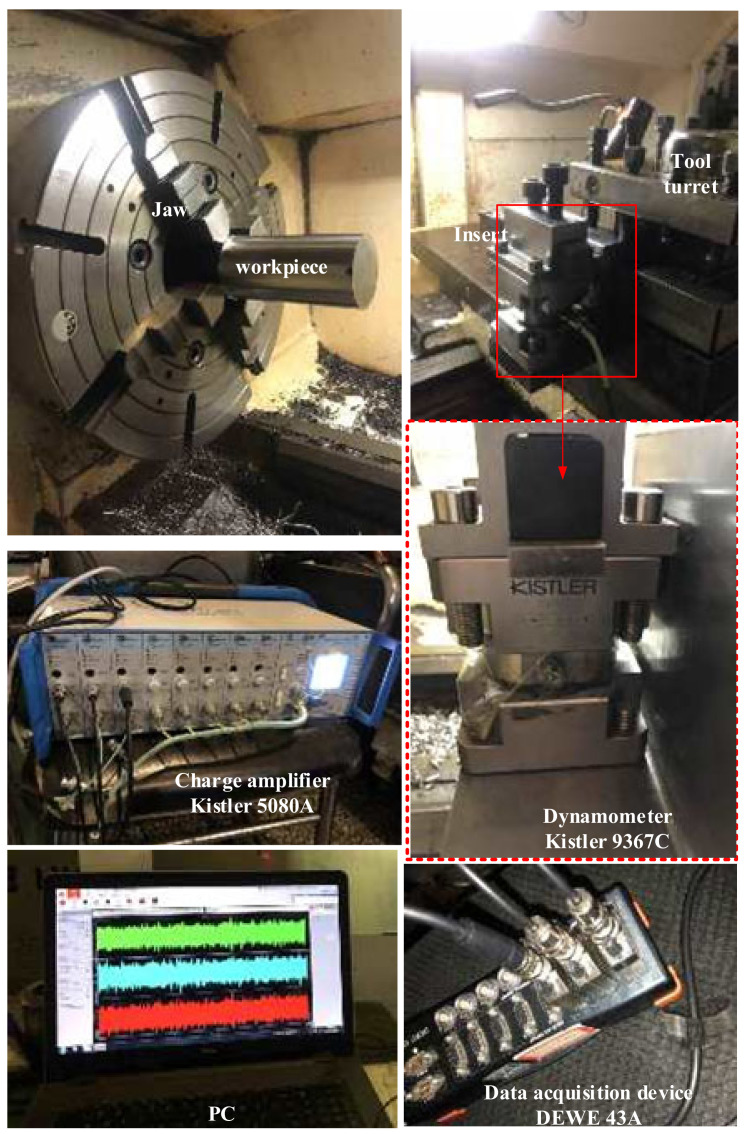
Photograph of the experimental setup.

The experimental setup is illustrated in Figure 12. The cutting force was measured using a Kistler9367C(Kistler Group, Winterthur, Switzerland) dynamometer. The dry turning process was performed using a CNC lathe (HK63, Bao Ji Machine tool Group Co. Ltd., Bao Ji, China), and the controller of the NC system was a FANUC series Oi mate TC. The workpiece was installed on the machine spindle and clamped using a four-jaw chuck. The dynamometer was installed on the turret of the lathe. The signal measured by the dynamometer was sent to a PC through USB via an amplifier (Kistler 8050A, Kistler Group, Winterthur, Switzerland) and a data collector (DEWE 43A, Dewesoft, Trbovlje, Slovenia). The experimental sampling frequency was 20 kHz. The measured turning force was displayed on the computer in real time.

According to Res. [34], the cutting force generated in the turning process was measured by the dynamometer in three directions, and the signal was transmitted to the amplifier, which transmits the signal to the data collector. The computer finally extracted single with software, and the force in the cutting process was measured and recorded in real time.

### 4.3. Cutting Force Model Validation

The tool and shank in Table 5 were selected for the turning experiments. As listed in Table 6, Tests 1–5 were performed to verify the consistency of the predicted and experimentally measured turning force at different cutting depths, whereas Tests 6–10 were performed to compare the experimental and predicted turning forces for a given feed rate *f*.

**Figure 13:**
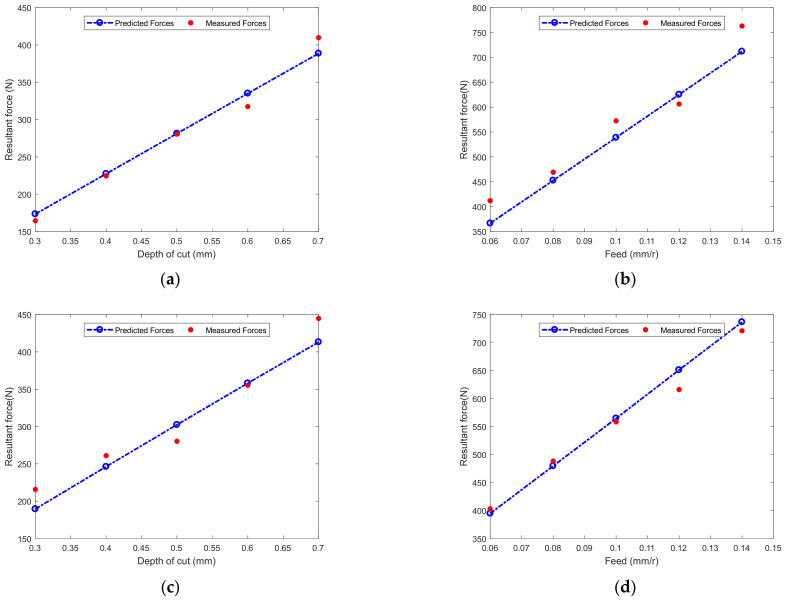
Comparison of measured and predicted cutting forces: (**a**) Tool No.1 and Tests 1–5, (**b**) Tool No.1 and Tests 6–10, (**c**) Tool No.2 and Tests 1–5, (**d**) Tool No.2 and Tests 6–10.

The prediction and experimental results are shown in Figure 13a–d. It can be observed that the predicted turning forces *F*^w^ are similar to those *F*^w^ obtained experimentally, it is clear that the developed cutting force model is effective. In Figure 13a,c, the turning force increases approximately linearly with the cutting depth. In Figure 13b,d, the turning force increases linearly with the feed rate. This can be explained that the area of undeformed material borne by the tool increases with the cutting depth and feed rate. This further shows that the machine tool power required is closely related to the cutting parameters.

In Figure 13a–d, the predicted forces were proportional to feed per revolution and cutting depth, and these results are consistent with the results reported in [26]. Sun and Duan [35] also observed the same trend in turning experimental when cutting particle reinforced metal matrix composites material. It is important for machining quality to select a suitable parameter in turning process. According to the changing trend of the predicted cutting force in Figure 13, the results can also optimize the cutting parameters.

Table 7 presents the relative errors between the predicted and experimental turning forces. The maximum relative errors for the total turning force predicted for Tool No. 1 and Tool No. 2 were −6.71% and −12.16%, respectively. Whereas, the average relative deviations were 4.78% and 4.64% respectively. The cutting process is very complex, some uncertain factors also effect cutting force. Thus, the relative error results show that the proposed model can be predicted accurately cutting force.

## 5. Conclusions

A new accurate analytical model for calculating the WNRA and WIA along the ACE was proposed in this study, and the effects of the workpiece radius size and cutting parameters on the WIA and WNRA were analyzed. The WIA and WNRA of the differential element cutting edge were applied to the turning force model, and an analytical model of the turning force was established. Turning experiments demonstrated that the proposed model is effective and accurate. Based on the results, the following conclusions can be drawn:

1. The velocity directions at differential points on the ACE are not parallel owing to the influence of the tool nose and rake angle. This contradicts the assumption of parallel cutting velocities in traditional machining.
2. During turning, the WNRA and WIA values for each differential element change continuously. A differential cutting edge with an edge inclination angle of zero is always present on the tool nose, implying that even when oblique machining is used in the turning, there is always an orthogonal cutting element.
3. For a given tool, when the workpiece radius is large, the variation range of cutting velocity direction at an element on the ACE is small. When the workpiece radius is small, the variation range of cutting velocity direction is large. The radius of the workpiece exerts a size effect on the WNRA and WIA.
4. The proposed analytical model of the turning force was verified through a series of experiments; the average deviation was less than 5%. The results indicates that the model is accurate and effective.

## Figures and Tables

**Table 1:** Simulation parameters.

Test No.	1	2	3	4	5
Rw (mm)	5	15	25	35	45
ap (mm)	3	3	3	3	3

**Table 2:** Normal rake angle and inclination angle in simulation.

Test No	1	2	3	4	5
γ (°)	3	8.2	16.2	21.9	26.6
λ (°)	1.1	5.4	9.8	14.1	17.4

**Table 3:** Chemical composition of GH4169.

Element	Ni	Cr	Nb	Mo	Ti	Al	Co	Mn	Cu	Si	C	Fe
wt %	52.15	19.26	5.03	3.03	1.08	0.56	0.5	0.22	0.1	0.26	0.052	17.75

**Table 4:** Typical mechanical properties at room temperature.

Tensile Strength (MPa)	Yield Strength (MPa)	Young’s Modulus (GPa)	Density (g·cm^−3^)	Poisson’s Ratio	Thermal Conductivity (W/m·K)
1430	1300	204	8.24	0.3	14.7

**Table 5:** Geometry of insert and tool holder.

Tool No.	Insert Type	Tool Holder Type	Rake Angle (°)	Inclination Angle (°)	Side Cutting Edge angle *k*_s_ (°)	End Cutting Edge angle *k*_e_ (°)	Nose *r* (mm)
1	VBET160408-NGF	SVJCR2525M16	12	8.2	93	52	0.8
2	CNMG120408-EF	DCLNR2525M12	14	6.1	95	5	0.8

**Table 6:** Cutting conditions.

Test No.	1	2	3	4	5	6	7	8	9	10
*f* (mm)	0.1	0.1	0.1	0.1	0.1	0.06	0.08	0.1	0.12	0.14
*a_p_* (mm)	0.3	0.4	0.5	0.6	0.7	1	1	1	1	1
Workpiece diameter(mm)	80	78	76	74	72	70	68	66	64	62

**Table 7:** Comparisons between experimental and predicted resultant forces *F*^w^.

	No.	1	2	3	4	5	6	7	8	9	10
Tool 1	Pre(N)	173.40	227.39	281.29	335.06	388.63	366.67	452.67	538.90	625.33	711.90
	Exp(N)	164.43	224.50	280.91	317.40	409.78	412.12	469.24	572.43	606.19	763.08
	Relative Error(%)	5.46	1.28	0.14	5.56	−5.16	−11.03	−3.53	−5.86	3.16	−6.71
Tool 2	Pre(N)	189.47	246.24	302.42	358.06	413.13	394.71	479.52	564.54	651.08	736.60
	Exp(N)	215.71	261.01	280.22	355.24	444.79	403.02	488.18	558.28	616.07	720.99
	Relative Error(%)	−12.16	−5.66	7.92	0.80	−7.12	−2.06	−1.77	1.12	5.68	2.16
